# Supplementary material for: Industrial scale high-throughput screening delivers multiple fast acting macrofilaricides
Source: Nat Commun. 2019 Jan 2;10:11. doi: 10.1038/s41467-018-07826-2 (PMC6315057; doi:10.1038/s41467-018-07826-2)
Supplement: Supplementary file 2 — Supplementary Information [file 41467_2018_7826_MOESM2_ESM.pdf]

**Industrial scale high-throughput screening delivers multiple fast acting macrofilaricides**

Clare et al.

Supplementary Figures 1 & 2

Supplementary Tables 1 to 6

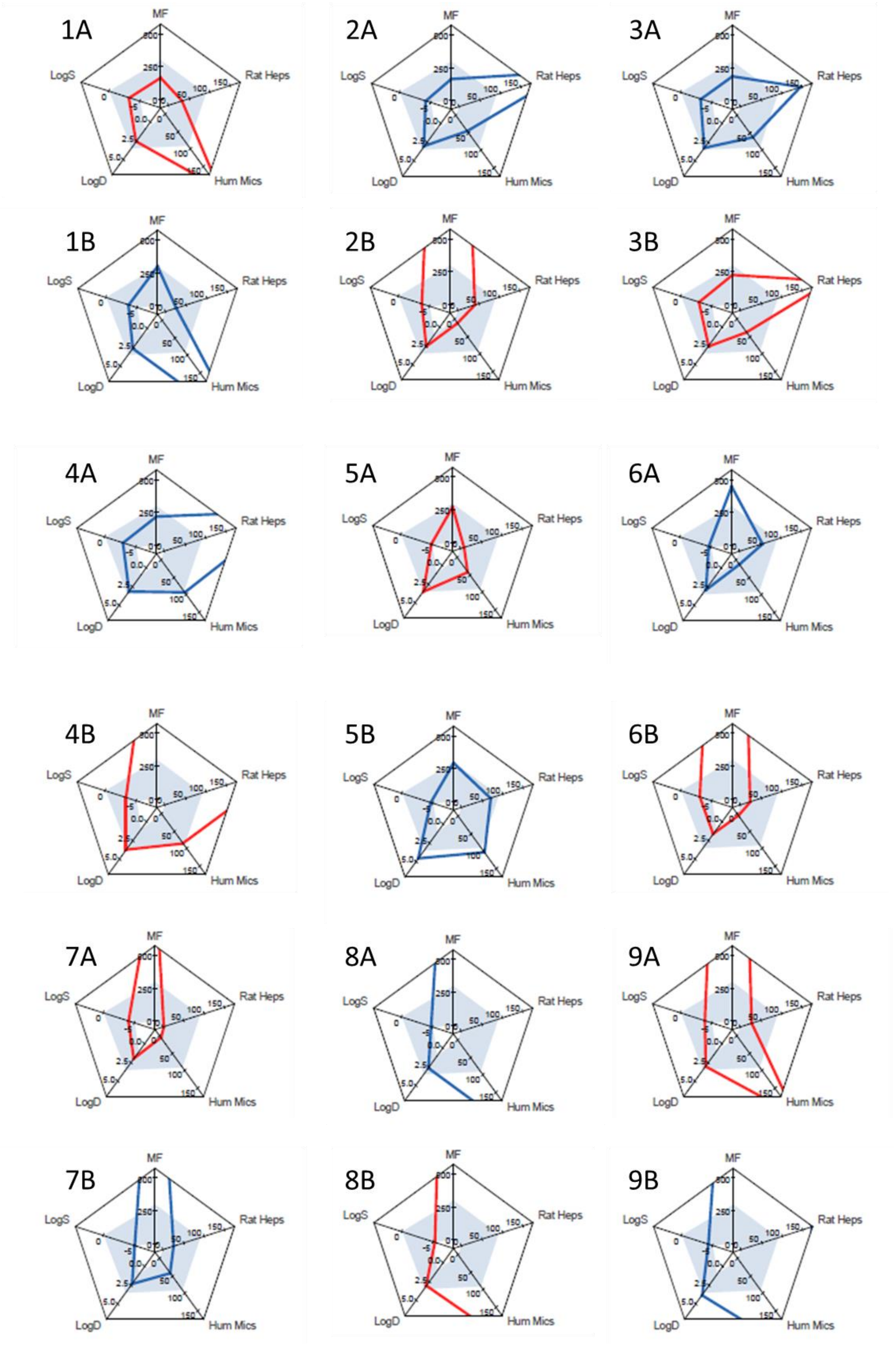


**Supplementary Figure 1| Anti-*Wolbachia* potency in the microfilarial assay and DMPK properties of the 18 selected hits**. Five axes in the radar plots represent: EC$_{50}$ values (nM) in the microfilarial assay (MF), rat hepatocytes clearance (µl min$^{-1}$1 x 10$^{6}$ cells $^{-1}$) (Rat Heps), human microsome clearance (µl min$^{-1}$ mg$^{-1}$) (Hum Mics), LogD$_{7.4}$ (LogD) and log value of aqueous solubility in pH7.4 PBS buffer (µM) (LogS). Desirable zone is in shaded grey.

A

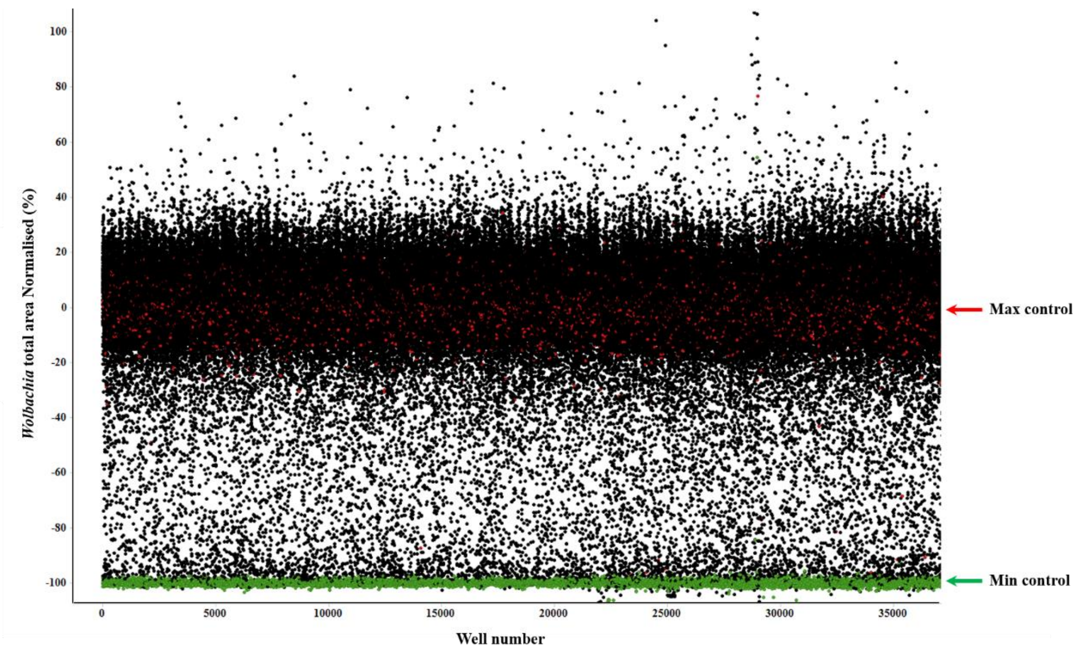


B

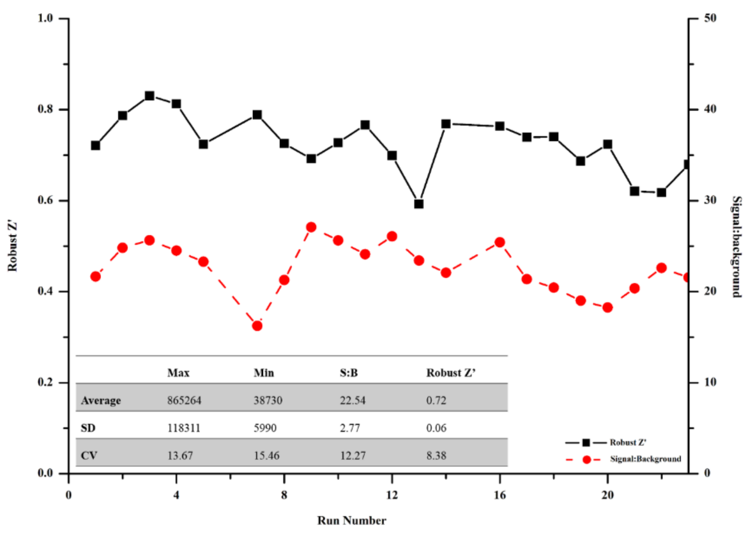


| | Max | Min | S:B | Robust Z' |
|---|---|---|---|---|
| Average | 865264 | 38730 | 22.54 | 0.72 |
| SD | 118311 | 5990 | 2.77 | 0.06 |
| CV | 13.67 | 15.46 | 12.27 | 8.38 |

**Supplementary Figure 2 A| Scatter plot analysis of the primary HTS campaign**. Data of single shot compounds (10 µM) for a representative set of plates (for clarity) (black circles), together with doxycycline controls (green circles) and vehicle controls (red circles). **B| Robust Z' and Signal to Background data**. Each data point represents 1 run on a single acumen® (i.e. 50-80 plates of data). Robust Z' (black squares and line) is consistently greater than 0.6 while the signal:background (red circle and dashed line) is around 23 throughout the screen. The screen statistics are tabulated and include averages, standard deviations (SD) and coefficient of variation (%).

**Supplementary Table 1| Triaging hits from the primary screen (20,000 to 12,000 compounds).** After the primary screen, removal of known antibacterials, PAINS, frequent hitters, known toxics, explosive risk, genotoxicity, reactive metabolites and unwanted chemical groups[1–5], resulted in a remaining 12,000 active compounds. All 12,000 compounds had several physicochemical properties calculated (LogD, aqueous solubility, human microsomal clearance, rat hepatocyte clearance and molecular weight) using algorithms developed by AstraZeneca. These properties were combined into a score through a summation of values following the specifications given in the table below. Between the threshold values shown, the score was calculated using linear interpolation.

| | Score = 1 | Score =0 |
|---|---|---|
| LogD7.4 | 1-3 | <0, >4 |
| Aqueous solubility (µM) | >50 | <10 |
| Human microsomal clearance (µl $min^{-1}$ $mg^{-1}$) | <30 | >90 |
| Rat hepatocyte clearance (µl $min^{-1}$ 1 x $10^6$ cells $^{-1}$) | <30 | >90 |
| Molecular weight | <350 | >500 |

**Supplementary Table 2| Clustering and further triaging of the hits from the primary screen (12,000 to 6,000 compounds).** The 12,000 compounds were clustered using ECFP4 fingerprints[6] and resulted in identifying 3,289 clusters. 1,157 singletons were found leaving 11,075 compounds in 2,132 clusters. The aim was to select the best set of clusters that had an optimal coverage of chemical space (cluster coverage) and mean property scores (LogD, aqueous solubility, human microsomal clearance, rat hepatocyte clearance and molecular weight). In order to achieve this a Pareto subset optimisation was performed using Pipeline Pilot software to select the final ~6,000 compounds for secondary screening. The mean properties of the subset of clusters selected through this method is shown below.

| LogD Score | Aqueous Solubility Score | Molecular Weight Score | Human Microsomal Clearance Score | Rat Hepatocyte Clearance Score | Cluster Coverage |
|---|---|---|---|---|---|
| 0.73 | 0.52 | 0.54 | 0.74 | 0.64 | 77% |

**Supplementary Table 3| Structures of the 18 selected compounds**

| Cluster | Code | Structure | AstraZeneca reference ID |
|---|---|---|---|
| | 1A | 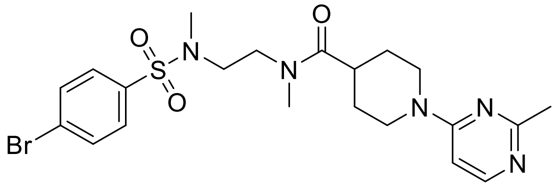 | SN0036849356 |
| 1 | 1B | 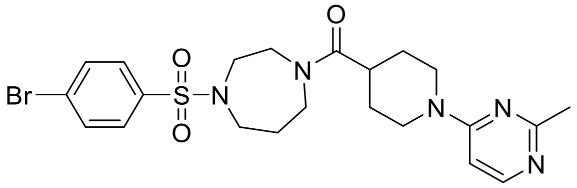 | SN0036438285 |
| | 2A | 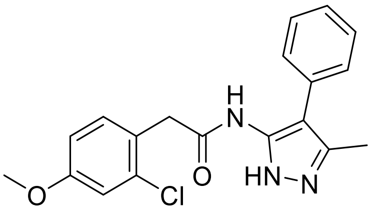 | SN1100209612 |
| 2 | 2B | 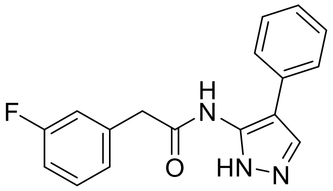 | SN1088675911 |
| | 3A | 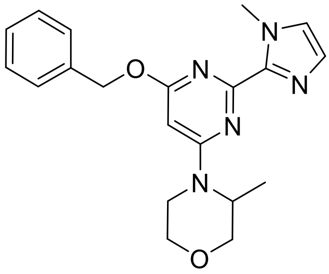 | SN1099356958 |
| 3 | 3B | 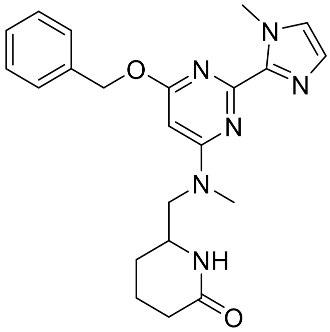 | SN1099254208 |
| 4 | 4A | 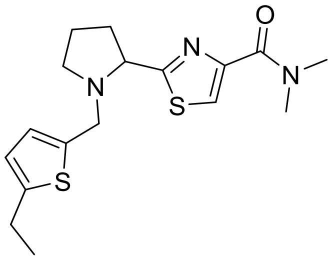 | SN1090550309 |

| | | | |
|---|---|---|---|
| | 4B | 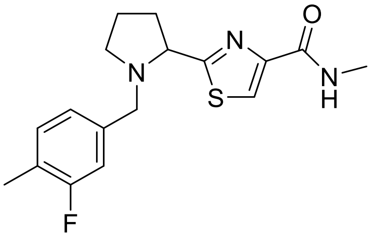 | SN1089649196 |
| | 5A | 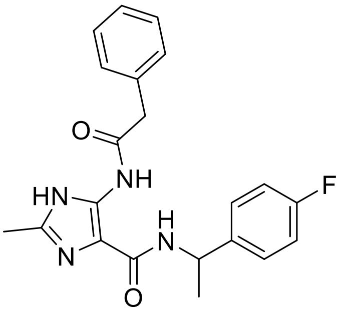 | SN1078536639 |
| 5 | 5B | 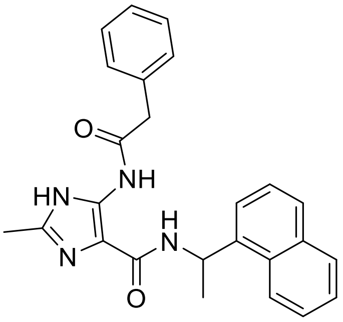 | SN1079789248 |
| | 6A | 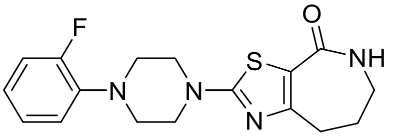 | SN0212745579 |
| 6 | 6B | 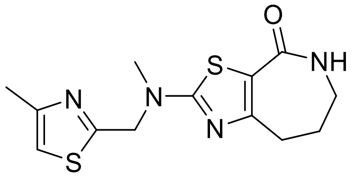 | SN0212747792 |
| | 7A | 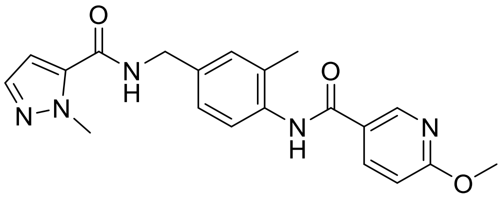 | SN1106450628 |
| 7 | 7B | 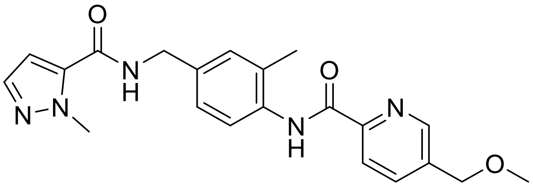 | SN1106449958 |
| 8 | 8A | 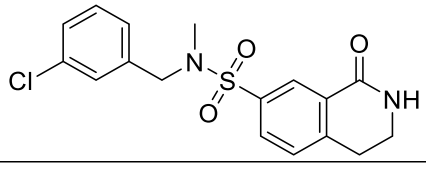 | SN1107250765 |

| | | | |
|---|---|---|---|
| | 8B | 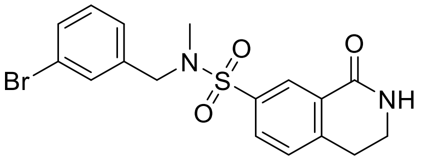 | SN1107099853 |
| | 9A | 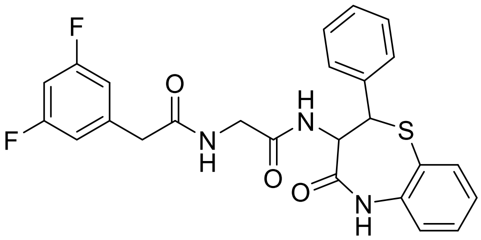 | SN0039632572 |
| 9 | 9B | 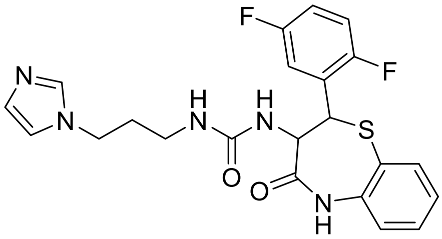 | SN0039778214 |

**Supplementary Table 4| Secondary screen Selection Score.** We adopted a similar approach to that of GlaxoSmithKline[7] to assign a selection score to each compound. We identified the best compounds through consideration of their potency, number of compounds in the cluster (using the 6,000 data set with ECFP6 fingerprints), measured human liver microsomal stability, measured aqueous solubility and measured Log D7.4. The total score is the summation of the individual scores and the maximum score a compound could take is 21. Further details are given in the table below.

| Mf Potency (nM) | Potency Score | Number of compounds in the cluster | Cluster Score | Human microsomal clearance (µl min$^{-1}$ mg$^{-1}$) | Human microsomal Score | Aqueous solubility (µM) | Solubility Score | LogD7.4 | LogD 7.4 Score |
|---|---|---|---|---|---|---|---|---|---|
| ≤200 | 6 | ≥30 | 3 | ≤10 | 6 | ≥1000 | 3 | ≥4 | 0 |
| >200 ≤300 | 5 | ≥15 ≤3 | 2 | >10 ≤15 | 3 | >500 ≤1000 | 2 | >3 ≤4 | Linear interpolation |
| >300 ≤500 | 4 | ≤3 | 1 | >15 ≤100 | 2 | >100 ≤500 | 1 | >1 <3 | 3 |
| >1000 ≤3000 | 3 | | | >100 ≤300 | 1 | ≤100 | 0 | >0 ≤3 | Linear interpolation |
| >3000 ≤5000 | 2 | | | ≤300 | 0 | | | ≤0 | 0 |
| >5000 | 1 | | | | | | | | |

**Supplementary Table 5| Screening results and chemical characteristics of the 18 selected compound**

| Cluster | Code | HTS $IC_{50}$ (nM) | HTS $IC_{50}$ mammalian toxicity (µM) | Confirmatory cell assay $EC_{50}$ (nM) | Mf $EC_{50}$ (nM) | Rat hepatocytes CLint (µl/min/1x$10^6$ cells) | Human microsome CLint (µl/min/mg) | LogD 7.4 | Aqueous solubility in pH7.4 PBS (µM) | Human plasma protein binding (%) | Purity (%) (LCMS/ HPLC) |
|---|---|---|---|---|---|---|---|---|---|---|---|
| 1 | 1A | 184.5 | > 30.63 | 192.0 | ~161 | 31.1 | 211.2 | 2.6 | 663.0 | 76.0 | 93.6 |
| | 1B | 111.2 | > 30.63 | 268.5 | ~296 | 24.8 | >300 | 2.7 | 217.0 | 77.0 | >95 |
| 2 | 2A | 2390.7 | > 30.63 | 241.7 | 164 | 262.8 | 41.2 | 3.1 | 71.0 | 97.9 | >95 |
| | 2B | 4368.3 | > 30.63 | 11313.0 | >5000 | 39.5 | 8.5 | 2.5 | 172.0 | 88.0 | >95 |
| 3 | 3A | 968.3 | > 30.63 | 662.1 | ~183 | 147.0 | 59.7 | 3.3 | 538.0 | 95.8 | 96.9 |
| | 3B | 510.5 | > 30.63 | 645.2 | ~223 | 218.6 | 35.5 | 2.5 | >1000 | 80.0 | >95 |
| 4 | 4A | 144.9 | > 30.63 | 41.0 | ~219 | >300 | 92.0 | 3.2 | >1000 | 92.2 | >95 |
| | 4B | 228.0 | > 30.63 | 66.0 | 1757 | >300 | 83.4 | 3.7 | 397.0 | 95.0 | >85 |
| 5 | 5A | 594.3 | > 30.63 | 296.0 | ~271 | 8.0 | 39.5 | 3.5 | 12.0 | 92.7 | >95 |
| | 5B | 158.1 | > 30.63 | 68.0 | 294 | 71.1 | 100.7 | 4.6 | 22.0 | 98.9 | >95 |
| 6 | 6A | 683.9 | > 30.63 | 2559.0 | 447 | 55.1 | 11.3 | 2.9 | 26.0 | 91.2 | >95 |
| | 6B | 594.3 | > 30.63 | 897.3 | >5000 | 22.9 | 3.5 | 1.6 | 756.0 | 60.0 | >95 |
| 7 | 7A | 1273.5 | 23.21 | 1137.0 | ~1161 | <1 | <3 | 2.0 | 100.0 | 99.7 | >95 |
| | 7B | 2483.1 | > 30.63 | 3074.0 | 2224 | 25.0 | 40.7 | 2.3 | 11.0 | 99.5 | >95 |
| 8 | 8A | 486.4 | > 30.63 | 515.3 | 2701 | >300 | >300 | 2.7 | 16.0 | 95.0 | >95 |
| | 8B | 534.6 | > 30.63 | 872.7 | >5000 | >300 | >300 | 3.1 | 6.0 | 96.6 | >95 |
| 9 | 9A | 181.1 | > 30.63 | 180.0 | >5000 | 28.0 | 227.9 | 3.0 | 105.0 | 88.0 | >95 |
| | 9B | 475.3 | > 30.63 | 482.6 | 2701 | 171.6 | >300 | 3.8 | 43.0 | 98.4 | >95 |

**Supplementary Table 6| Primer design for the qPCR analysis in the tertiary screen.** The primers are based on either *Brugia malayi wsp* (surrogate for *Wolbachia* quantification) or *Brugia malayi gst* (surrogate for nematode quantification).

| Primer name | Sequence position | Sequence | Product size | Melting temperature | cDNA accession number |
|---|---|---|---|---|---|
| BMWSP_forward | 456 | 5'CCCTGCAAAGGCACAAGTTATTG 3' | 117 | 65 | AJ252061 |
| BMWSP_reverse | 572 | 5' CGAGCTCCAGCAAAGAGTTTAATTT 3' | 117 | 63 | |
| BMGST_forward | 1368 | 5' GAGACATCTTGCTCGCAAAC 3' | 264 | 59 | Y12788 |
| BMGST_reverse | 1632 | 5' ATCACGGACGCCTTCACAG 3' | 264 | 59 | |

Supplementary References


1. Rishton, G. M. Reactive compounds and in vitro false positives in HTS. *Drug Discov. Today* **2,** 382–384 (1997).

2. Hann, M. *et al.* Strategic pooling of compounds for high-throughput screening. *J. Chem. Inf. Comput. Sci.* **39,** 897–902 (1999).

3. Oprea, T. I. Property distribution of drug-related chemical databases. *J. Comput. Aided. Mol. Des.* **14,** 251–64 (2000).

4. Muegge, I. Selection criteria for drug-like compounds. *Med. Res. Rev.* **23,** 302–321 (2003).

5. Baell, J. B. & Holloway, G. A. New Substructure Filters for Removal of Pan Assay Interference Compounds (PAINS) from Screening Libraries and for Their Exclusion in Bioassays. *J. Med. Chem.* **53,** 2719–2740 (2010).

6. Rogers, D. & Hahn, M. Extended-Connectivity Fingerprints. *J. Chem. Inf. Model.* **50,** 742–754 (2010).

7. Calderón, F. *et al.* An invitation to open innovation in malaria drug discovery: 47 quality starting points from the TCAMS. *ACS Med. Chem. Lett.* **2,** 741–746 (2011).
