# Supplementary material for: Industrial scale high-throughput screening delivers multiple fast acting macrofilaricides
Source: Nat Commun. 2019 Jan 2;10:11. doi: 10.1038/s41467-018-07826-2 (PMC6315057; doi:10.1038/s41467-018-07826-2)
Supplement: Supplementary file 3 — Description of Additional Supplementary Files [file 41467_2018_7826_MOESM3_ESM.pdf]

Description of Additional Supplementary Files

File Name: Supplementary Data 1
Description: Chemical characterisation of the 18 selected hits
